# GABAergic synapses onto SST and PV interneurons in the CA1 hippocampal region show cell-specific and integrin-dependent plasticity

**DOI:** 10.1038/s41598-023-31882-4

**Published:** 2023-03-28

**Authors:** Patrycja Brzdąk, Katarzyna Lebida, Marcin Wyroślak, Jerzy W. Mozrzymas

**Affiliations:** Department of Biophysics and Neuroscience, Wroclaw Medical University, 50-367, Wroclaw, Poland

**Keywords:** Neuroscience, Cellular neuroscience, Learning and memory, Molecular neuroscience, Synaptic transmission

## Abstract

It is known that GABAergic transmission onto pyramidal neurons shows different forms of plasticity. However, GABAergic cells innervate also other inhibitory interneurons and plasticity phenomena at these projections remain largely unknown. Several mechanisms underlying plastic changes, both at inhibitory and excitatory synapses, show dependence on integrins, key proteins mediating interaction between intra- and extracellular environment. We thus used hippocampal slices to address the impact of integrins on long-term plasticity of GABAergic synapses on specific inhibitory interneurons (containing parvalbumin, PV + or somatostatin, SST +) known to innervate distinct parts of principal cells. Administration of RGD sequence-containing peptide induced inhibitory long-term potentiation (iLTP) at fast-spiking (FS) PV + as well as on SST + interneurons. Interestingly, treatment with a more specific peptide GA(C)RRETAWA(C)GA (RRETAWA), affecting α5β1 integrins, resulted in iLTP in SST + and iLTD in FS PV + interneurons. Brief exposure to NMDA is known to induce iLTP at GABAergic synapses on pyramidal cells. Intriguingly, application of this protocol for considered interneurons evoked iLTP in SST + and iLTD in PV + interneurons. Moreover, we showed that in SST + cells, NMDA-evoked iLTP depends on the incorporation of GABA_A_ receptors containing α5 subunit to the synapses, and this iLTP is occluded by RRETAWA peptide, indicating a key role of α5β1 integrins. Altogether, our results revealed that plasticity of inhibitory synapses at GABAergic cells shows interneuron-specificity and show differences in the underlying integrin-dependent mechanisms. This is the first evidence that neuronal disinhibition may be a highly plastic process depending on interneuron type and integrins’ activity.

## Introduction

Activity-dependent synaptic plasticity shaping the functioning of neural networks is nowadays recognized as a key mechanism of engram formation and therefore of memory encoding and learning^1^. For long, most studies have focused on the plasticity of glutamatergic synapses tacitly assuming that the inhibitory synapses were largely invariant and, consequently, memory and learning were primarily attributed to plastic changes at excitatory synapses. In the hippocampal formation, GABAergic interneurons represent only 10–15% of the neuronal population but due to their extraordinary morphological and functional diversity (only in CA1 at least 23 subtypes) they are able to exert a precise control of cellular and network functioning^2–4^. Interneurons are subdivided primarily according to their morphology, electrophysiological and neurochemical properties as well as innervation pattern of the principal cells. Among various INs types, defined by expression of the molecular markers, somatostatin (SST) and parvalbumin (PV)-positive cells form two broad interneurons classes. PV expressing interneurons contact perisomatic area of principal cells while SST interneurons target pyramidal cells (PCs) dendrites. Importantly, besides pyramidal cells, PV interneurons innervate other types of inhibitory cells e.g. oriens lacunosum-moleculare (O-LM) and bistratified SST-positive interneurons but mainly other PV-positive INs^5,6^. It is important to note that specific subgroups of interneurons e.g. VIP + INs target other interneurons (PV + and SST +) giving rise to neuronal disinhibition, one of the key phenomena involved in regulation of excitation/inhibition balance^7^. Recent studies, using genetically targeted recordings, enabled an insight into the mechanisms whereby distinct interneurons participate in the formation of hippocampal memory^5,6^. This expanding knowledge on GABAergic inhibition encouraged a more “holistic” approach considering additionally plasticity at GABAergic synapses and its interplay with the glutamatergic drive^8–11^. Consequently, in the past decade or so, GABAergic inhibition was shown to undergo many forms of plasticity and the underlying molecular mechanisms start to be unraveled^8^. In particular, we have recently shown that stimulation of transmembrane adhesion molecules, integrins with specific peptides induced different types of GABAergic plasticity in the hippocampus^12^ while Kawaguchi and Hirano (2006) reported that α3β1 integrin suppressed long-term potentiation at inhibitory synapses on the cerebellar Purkinje neurons. Interestingly, integrins were also found to be involved in the plasticity of glycinergic inhibition^13^. Moreover, a key impact of these adhesion proteins has been implicated in spatial memory, although this role was attributed primarily to the plasticity of glutamatergic transmission^14,15^.

A heterosynaptic NMDAR-dependent GABAergic plasticity have been described in the hippocampus^12,16,17^ and in the cerebral cortex^18^. It has been proposed that during iLTP induced by a brief NMDA application a trapping and immobilization of GABA_A_ receptors (GABA_A_Rs) in the synaptic area is taking place^12,17^. Davenport et al., (2021) have shown that a form of TBS-induced (theta burst stimulation-induced) plasticity at glutamatergic synapses is associated with translocation of extrasynaptic α5 subunit-containing GABA_A_Rs into the inhibitory synapses. Notably, investigations addressing the roles of integrins and the heterosynaptic NMDA-induced plasticity were based on recordings of the inhibitory postsynaptic currents (IPSCs) mostly from pyramidal neurons while plasticity of phasic inhibition onto interneurons still awaits investigation. Considering the aforementioned importance of interneuron-interneuron inhibitory transmission and involvement of interneurons in memory formation, it seems appealing to explore the plasticity of GABAergic synaptic currents in the inhibitory neurons. To this end, we tested the impact of peptides affecting various types of integrins on GABAergic plasticity in parvalbumin-containing (PV + INs) and somatostatin-containing (SST + INs) interneurons, known to innervate distinct parts of pyramidal neurons (PV + INs—perisomatic, SST + INs—distal dendrites^4^) and to differently shape the network activity and memory formation^5,6,19–21^. We report that administration of distinct integrin-interfering peptides induce cell-specific GABAergic plasticity. In addition, we found that application of protocol inducing NMDAR-dependent plasticity^12,16,17^ gave rise to opposite plastic changes of GABAergic transmission in PV + and SST + interneurons.

## Results

### RGD peptide differentially modulate GABAergic synaptic transmission onto fast-spiking and non-fast spiking PV + interneurons

The Arg-Gly-Asp (RGD) sequence is present in numerous ECM proteins like fibronectin or laminin and is recognized by many integrins subtypes such as αvβ3, αvβ5, α5β1, α8β1, αvβ6, αvβ8, αvβ1, including those found in neurons^22,23^. Thus, herein, we used this peptide (see Table 1) to address the role of RGD-binding integrins in the plasticity of GABAergic transmission onto PV + and SST + interneurons.

**Figure 1:**
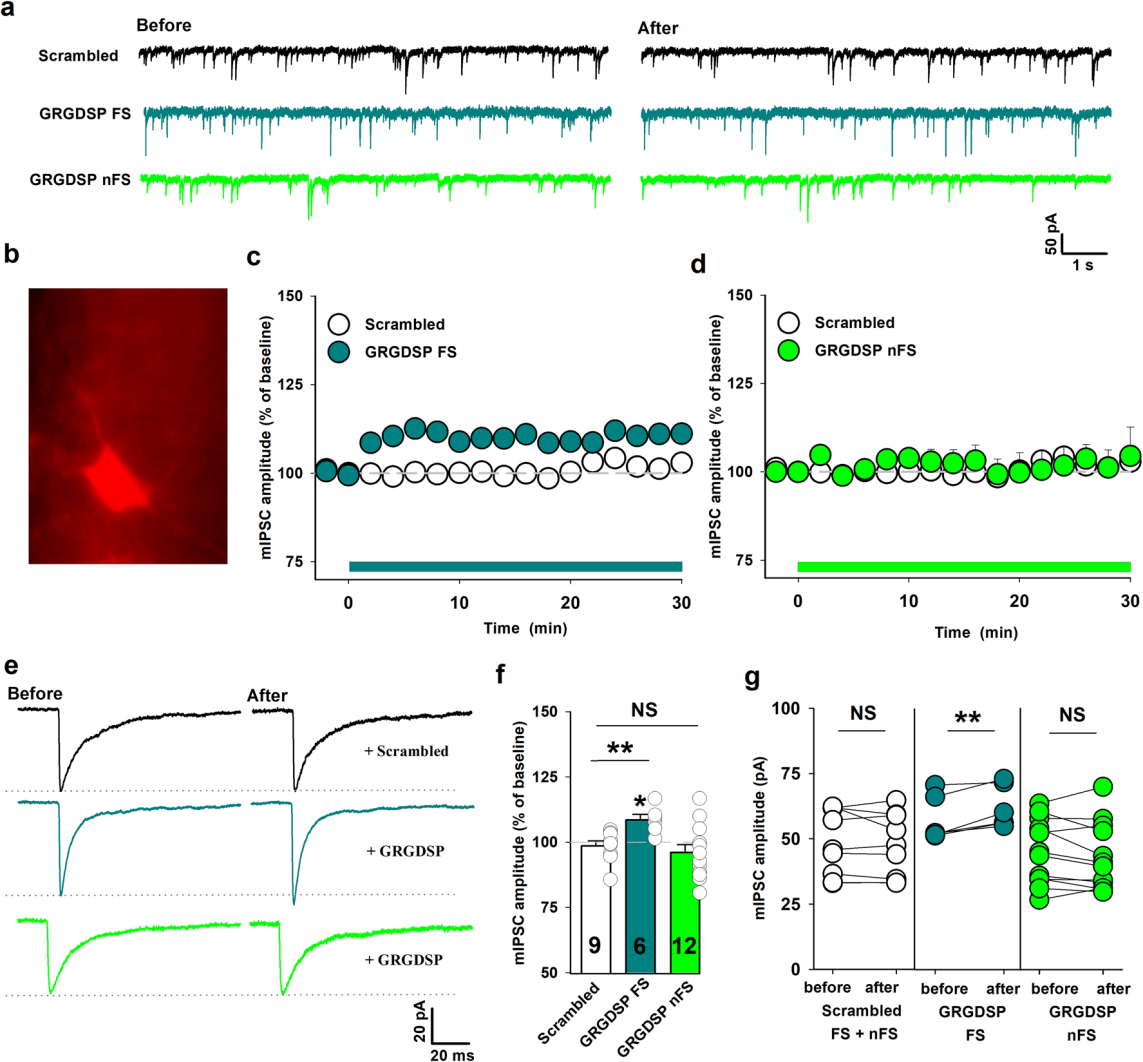
GRGDSP peptide induces potentiation of mIPSCs in FS but not in nFS PV + interneurons. (**a**) Examples of raw mIPSC traces recorded at baseline (left) and 16–18 min. after bath-application of GRGDSP (0.5 mM) or scrambled (GRADSP, 0.5 mM) peptide (right). Traces before and after scrambled peptide administration are presented with black for the control group (including nFS INs and FS INs, shown only for nFS INs). Sample traces for GRGDSP treatment are presented with dark green for FS INs and with light green for nFS INs. (**b**) Representative fluorescence image showing tdTomato-positive PV cell. (**c**–**d**) Time course of mIPSC amplitude before and after application of GRGDSP or scrambled peptide recorded from FS and nFS PV + INs. Data are binned into 2-min time bins. Timing of peptide administration is indicated by horizontal green line. (**e**) Averaged traces before and after integrin control peptide application (upper, for nFS PV + INs) and for GRGDSP-treated FS PV + (middle) and nFS PV + INs (lower). For groups describing treatment with peptides, averaging was performed for time window 16–18 min. after peptide administration. Dotted lines indicate mean values of mIPSC amplitude upon baseline recordings. (**f**) Summary plots of mIPSC amplitude changes (relative to baseline) after 16–18 min. window in control conditions (treatment with scrambled peptide GRADSP for FS and nFS INs) and GRGDSP-treated slices for FS PV + and nFS PV + INs groups. Asterisk above bin describing GRGDSP FS INs group indicates significant increase in amplitude relative to baseline. Additional tests: *t*-test: FS vs CTR, *p* = 0.005, Mann–Whitney Rank Sum Test: nFS vs scrambled peptide, *p* = 0.536. (**g**) Absolute values of mean mIPSC amplitudes in individual experiments before and after administration of peptides described below graphs for specific interneuron types (scrambled peptide for FS and nFS INs). Paired *t*-test was applied for these data. Note increased mIPSC amplitude after GRGDSP application in FS PV + INs. Average mIPSC amplitudes before and after treatment for an individual cell are presented as two circles connected with bar. Numbers on the bars refer to the number of recordings. *p < 0.05; **p < 0.01; NS, non-significant.

To characterize postsynaptically the GABAergic transmission and its plasticity, we measured GABA_A_ receptor-mediated miniature inhibitory postsynaptic currents (mIPSCs, Fig. 1a) in the whole-cell configuration from CA1 PV + cells in slices from cross-bred animals (PV-Cre and Ai14, Cre-dependent reporter strain, see “Materials and Methods”, Fig. 1b). Since PV-expressing interneurons are not a homogenous group, even in the region of our interest, we decided to divide them into 2 subgroups according to their location and firing pattern: fast-spiking (FS) PV + interneurons located in stratum pyramidale and in stratum oriens, non-fast spiking (nFS) PV + interneurons placed in stratum pyramidale and in stratum radiatum. Such a division to FS and nFS INs reflects the difference in input received by PV + interneurons and their participation in different neuronal circuits^4,24^.

Interestingly, we observed that after addition of GRGDSP peptide (hereafter referred to as RGD, 0.5 mM), mIPSC amplitude measured from FS PV + interneurons was slightly but significantly potentiated (108 ± 2%, n = 6, relative to baseline at 16–18 min. window after RGD administration; before RGD: 57.5 ± 3.6 pA, after RGD: 62.3 ± 3.3 pA, *p* = 0.009, Fig. 1c, e–g). The amplitude of mIPSCs remained potentiated after RGD washout (data not shown), confirming stable induction of this form of plasticity. However, in contrast to FS cells, we did not observe any significant change in mIPSC amplitude recorded from nFS interneurons (96 ± 3%, n = 12, relative to baseline, before RGD: 45 ± 3.6 pA, after RGD: 43.3 ± 3.7 pA, *p* = 0.229, Fig. 1d, e–g).

The potentiating effect observed for FS interneurons (Fig. 1 c, e–g) was specific to RGD, because when we used the scrambled peptide (GRADSP, 0.5 mM), no significant change in mIPSC amplitude was observed (GRADSP-scrambled pooled data for FS and nFS INs: 98 ± 2%, before 48.5 ± 4.2 pA, after 47.6 ± 4.1 pA, n = 9, *p* = 0.499, Fig. 1c–g). Neither RGD nor scrambled peptide significantly altered the mIPSC frequency in any of the considered groups of PV + interneurons (GRADSP-scrambled peptide pooled data for FS and nFS: 102 ± 4%, n = 8, *p* = 0.376; RGD: 103 ± 9%, n = 6, *p* = 0.680; 104 ± 8.5%, n = 12, *p* = 0.776 for FS and nFS respectively, relative to baseline, Supplementary Fig. 1a). Since scrambled peptide did not affect either mIPSC amplitude or frequency in the two PV + INs types, in the above statistics the control data were pooled.

We have additionally checked whether RGD administration affected the mIPSC time course in PV + INs but in none of considered subgroups this peptide caused any effect on either decay or onset kinetics of these currents (data not shown).

Altogether, we found that administration of the RGD peptide results in a stable form of inhibitory plasticity manifested by a relatively small but significant enhancement of mIPSCs amplitude in FS but not in nFS PV + interneurons.

### RGD peptide increases the efficacy of GABAergic synaptic transmission onto SST + interneurons

**Figure 2:**
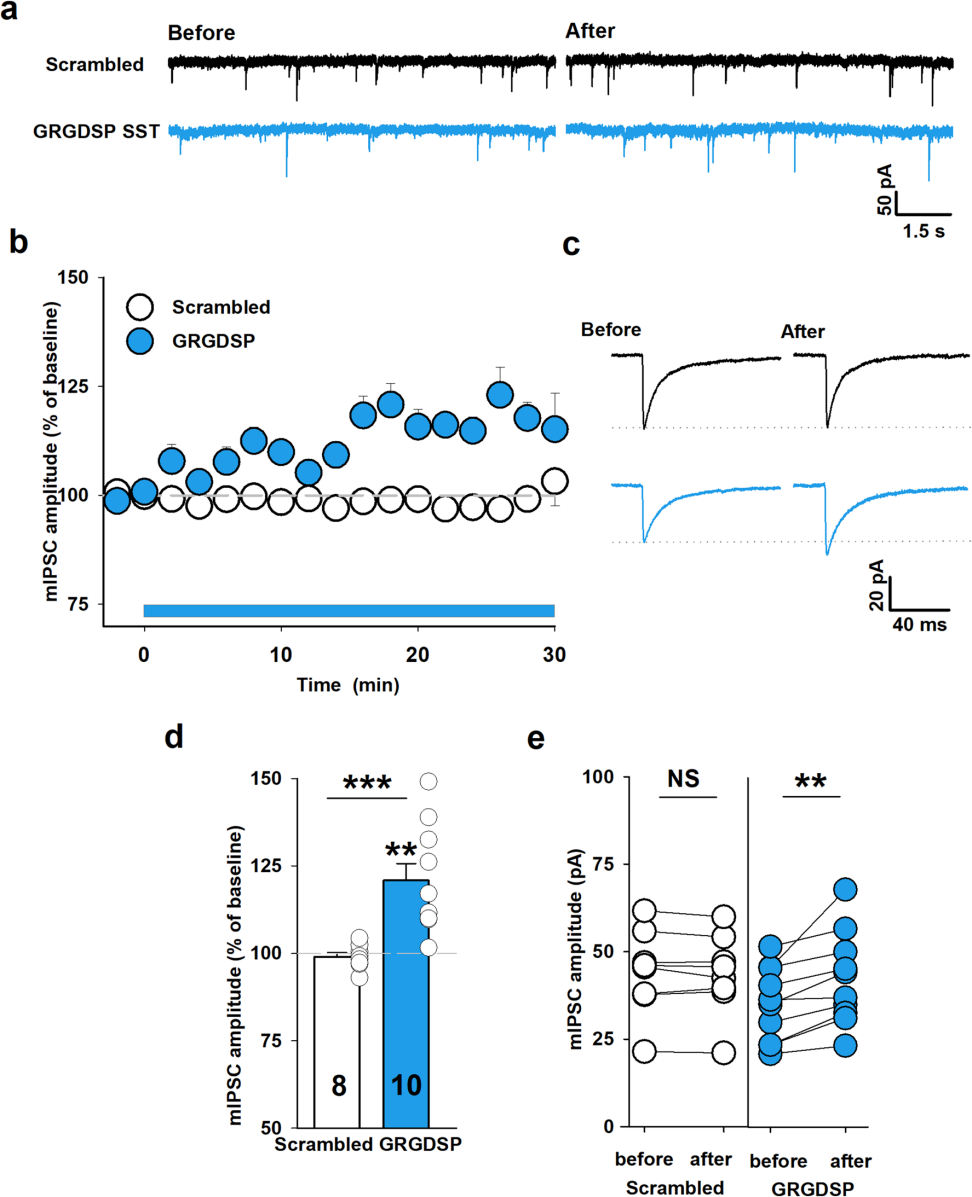
GRGDSP peptide increases the efficacy of GABAergic transmission in SST + INs. (**a**) Representative mIPSC traces recorded upon baseline recordings (before peptide application, left) and after 16–18 min. (right) scrambled peptide (GRADSP, 0.5 mM, black) or GRGDSP (0.5 mM, blue) peptide administration. (**b**) Time course of mIPSC amplitude in the presence of GRGDSP peptide and in control conditions (scrambled). Timing of peptides administration is described by the blue horizontal bar. Note that GRGDSP peptide increases the mIPSC amplitude. (**c**) Averaged traces obtained from baseline recordings (before peptide application, left) and after 16–18 min. (right) of scrambled (upper, black) or GRGDSP (lower, blue) peptide administration. Dotted lines indicate mean values of mIPSC amplitude upon baseline recordings. (**d**) Statistics for mIPSC amplitude changes after 16–18 min. (relative to baseline) in control conditions (scrambled peptide) and in GRGDSP-treated slices for SST + INs. Asterisk above bin describing GRDGSP group indicates significant increase in amplitude relative to baseline. Additional test: Mann–Whitney Rank Sum Test, GRGDSP vs. scrambled peptide, *p* < 0.001. (**e**) Absolute values of mean mIPSC amplitudes in individual experiments before and after administration of peptides described below graphs. Scrambled peptide: paired *t*-test, GRGDSP: Wilcoxon Signed Rank Test. **p < 0.01, ***p < 0.001; NS, non-significant.

In the next step, we tested the impact of RGD peptide administration on mIPSCs measured from SST + interneurons located in the stratum oriens area of the CA1 region (Fig. 2a). To address this issue specifically in SST + INs, we have examined tdTomato-expressing cells in the SST-Cre mouse line. Bath application of RGD peptide (0.5 mM) induced a clear potentiation of mIPSC amplitude that developed over 30 min during RGD treatment (RGD: 121 ± 5% at 16–18 min. window after RGD administration, compared to baseline, n = 10, before RGD: 35.3 ± 3.4 pA, after RGD: 42.4 ± 4.2 pA, *p* = 0.002, Fig. 2a–e). We did not observe any significant change of mIPSC amplitude in the presence of the scrambled peptide (GRADSP-scrambled: 99 ± 1%, n = 8, before: 44.1 ± 4.3 pA, after: 43.6 ± 4.1 pA, n = 8, *p* = 0.342, Fig. 2a–e) confirming thus the specificity of RGD action. No significant changes were found in the relative mean frequency of mIPSCs after RGD peptide administration compared to baseline (GRADSP-scrambled: 85 ± 5%, n = 8, *p* = 0.119; RGD: 111 ± 10%, n = 10, *p* = 0.152, Supplementary Fig. 1b). As in the case of PV + INs, RGD administration had no effect on decay or onset kinetics of mIPSCs (data not shown).

Thus, similar to FS PV + cells, application of RGD peptide induces potentiation of mIPSC amplitude in SST + interneurons without affecting the time course of these currents.

### Inhibition of α5β1 integrins differently affects GABAergic plasticity at PV + and SST + interneurons

**Figure 3:**
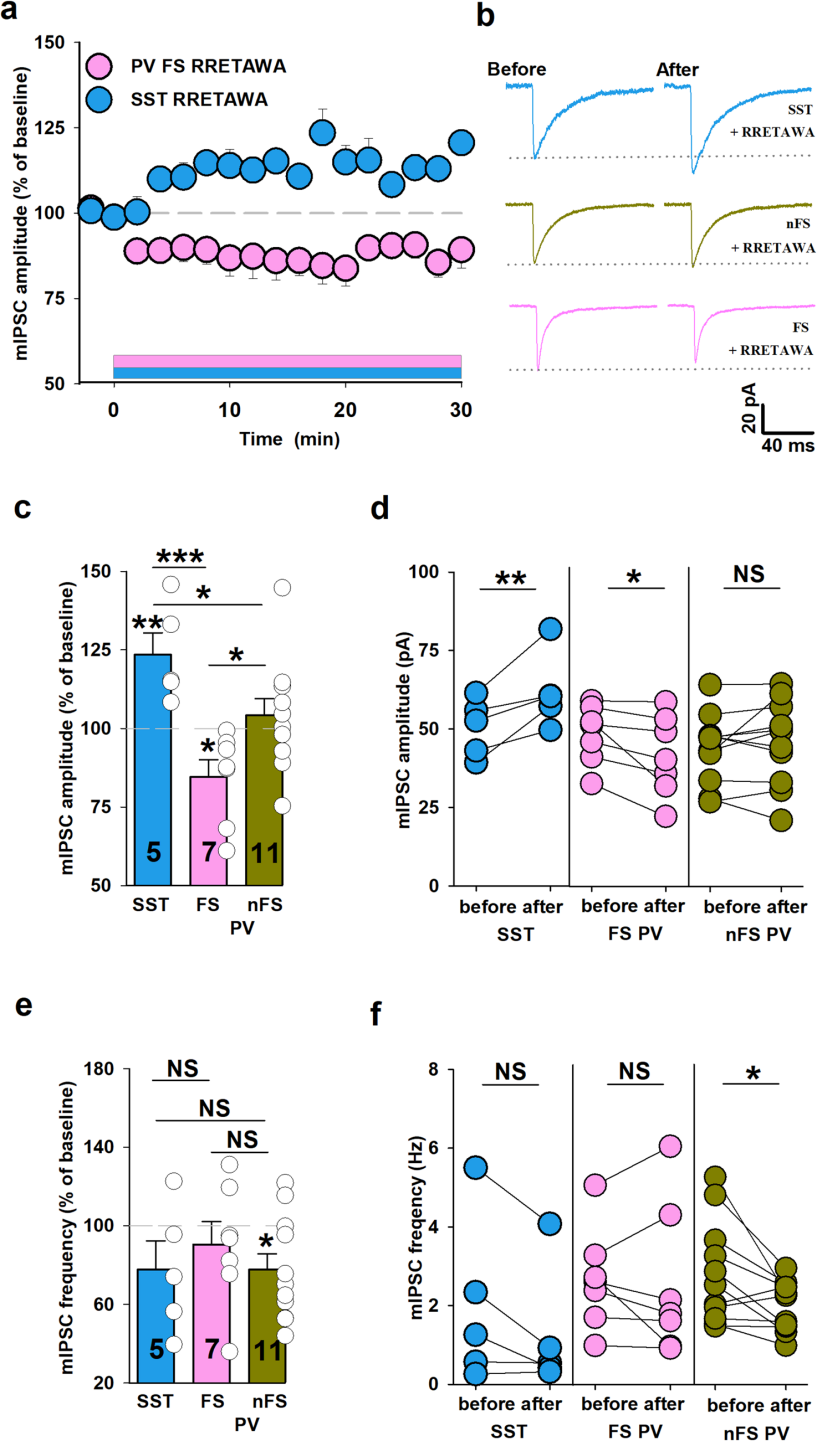
Inhibition of α5β1 integrins differently affects GABAergic transmission in SST + and PV + interneurons. (**a**) Averaged time courses of mIPSC amplitude in the presence of RRETAWA peptide (0.15 mM) in SST + INs (blue circles) and FS PV + INs (pink circles) groups. Timing of peptides administration is shown by the blue and pink horizontal bar. (**b**) Representative averaged traces upon baseline recordings (before) and 16–18 min. after RRETAWA administration (SST + —blue, upper, nFS PV + —green, middle and FS PV + INs —pink, lower). The dotted line shows the mean value of mIPSC baseline amplitude. (**c**) Summary plots of mIPSC amplitude changes after 16–18 min. (relative to baseline) of RRETAWA administration in SST + , FS PV + and nFS PV + INs. Note that inhibition of α5β1 integrin-dependent adhesion affects GABAergic transmission in SST + and FS PV + INs in an opposing manner. Asterisks above bins show significant change in amplitude relative to baseline. Additional test: one-way ANOVA with Holm-Sidak post hoc test; F(2,19) = 8.41, *p* = 0.002. (**d**) Absolute values of mean mIPSC amplitudes in individual experiments before and after administration of RRETAWA peptide in groups described below graphs. Paired *t*-test was used for these data. (**e**) Effect of inhibition of α5β1 integrin on mIPSC frequency after 16–18 min. application of RRETAWA peptide (relative to baseline) in SST + , FS PV + INs and nFS PV + groups. Asterisk above bin in nFS INs group indicates significant decrease in frequency relative to baseline. Additional test: one-way ANOVA; F(2,19) = 0.47, *p* = 0.634. (**f**) Absolute values of mean mIPSC frequency in individual recordings before and after administration of RRETAWA peptide in SST + , FS PV + and nFS PV + INs. *p < 0.05, **p < 0.01, ***p < 0.001; NS, non-significant.

To further characterize the role of integrins in GABAergic synaptic plasticity, we used a cyclic GA(C)RRETAWA(C)GA peptide (RRETAWA), which blocks α5β1-mediated cell adhesion to fibronectin ^25–27^. Bath application of RRETAWA (0.15 mM) significantly reduced mIPSC amplitude measured from FS interneurons (RRETAWA: 85 ± 5%, n = 7, at 16–18 min. window after peptide administration, relative to baseline, before RRETAWA: 47.8 ± 3.5 pA, after RRETAWA: 41 ± 4.8 pA, *p* = 0.035, Fig. 3a–d), but it was ineffective in the case of nFS cells (RRETAWA 104 ± 5%, n = 11, *p* = 0.29, before: 43.3 ± 3.3 pA, after: 45.2 ± 4 pA, *p* = 0.37; Fig. 3b–d). Interestingly, in the case of SST + INs, administration of RRETAWA increased mIPSC amplitude (RRETAWA 123 ± 7%, n = 5, before: 50.5 ± 4.1 pA, after: 61.9 ± 5.3 pA, *p* = 0.023; Fig. 3a–d). Application of the cyclic peptide did not affect kinetic properties of mIPSC (rise time, decay) in any of considered groups of interneurons. The effect of RRETAWA on mIPSC amplitude after the peptide washout was maintained (data not shown). We have additionally checked the impact of RRETAWA on mIPSCs frequency and, in contrast to our observations for RGD peptide (Supplementary Fig. 1a,b), treatment with RRETAWA resulted in a change of this parameter. In the case of nFS cells RRETAWA reduced the mIPSC frequency from 2.78 ± 0.39 Hz to 1.97 ± 0.19 Hz (n = 11, *p* = 0.022, Fig. 3e–f) but no significant effect was observed for the FS PV + INs (before: 2.65 ± 0.48 Hz, after: 2.52 ± 0.71 Hz, n = 7, *p* = 0.725, Fig. 3e–f). In the case of SST + interneurons a trend toward mIPSC frequency reduction upon RRETAWA treatment was apparent, but this effect did not reach statistical significance (before: 1.26 ± 0.71 Hz, after: 0.73 ± 0.17 Hz, n = 5, *p* = 0.087, Fig. 3e–f). The altered mIPSC frequency may suggest a presynaptic effect or change in synapse number, although the latter possibility is less probable within the considered time scale of the effect onset.

Altogether, we show that α5β1 integrins exert cell-specific modulation of GABAergic plasticity and, most interestingly, this effect on mIPSC amplitude is opposite in the case of FS PV + and SST + INs.

### Opposite signs of NMDAR-dependent inhibitory plasticity in PV + and SST + INs

**Figure 4:**
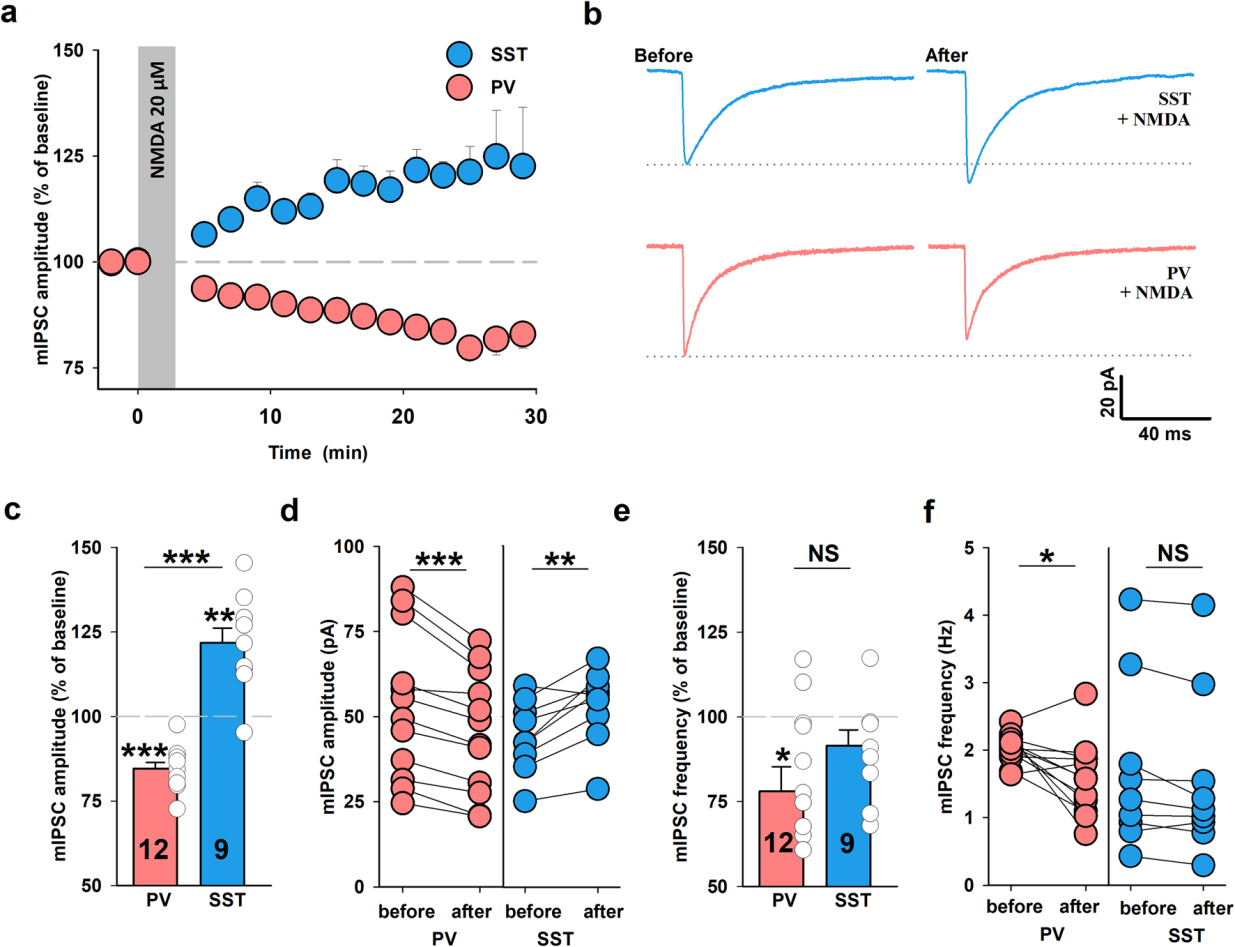
Brief NMDA treatment induces opposite sign plasticity in SST + and PV + INs. (**a**) Time courses of mIPSC amplitude after a brief NMDA exposure (3 min., 20 μM) in SST + (blue) and PV + INs (coral). The administration of NMDA is marked as the gray bar. Note that NMDA application significantly increased mIPSC amplitude in SST + INs (iLTP) and decreased mIPSC amplitude in PV + INs (iLTD). (**b**) Representative averaged mIPSC traces before and 16–18 min. after the end of NMDA treatment in SST + and PV + INs. Dotted line shows the mean value of mIPSC baseline amplitude. (**c**) Statistics for iLTD and iLTP magnitude 16–18 min. after the end of NMDA application (relative to baseline) in PV + and SST + INs. Asterisks above bins indicate significant change in amplitude relative to baseline. Additional test: Mann–Whitney Rank Sum Test, *p* < 0.001. (**d**) Average mIPSC amplitude measured from a single cell before and after NMDA stimulation in PV + INs and SST + INs. SST: paired *t*-test; PV: paired *t*-test with Wilcoxon Signed Rank Test. (**e**) The impact of NMDA administration on mIPSC frequency 16–18 min. after the end of plasticity induction in PV + and SST + INs (relative to baseline). Asterisk shows decrease in mIPSC frequency after iLTD induction. Additional test:* t*-test, *p* = 0.205. (**f**) Mean value of mIPSC frequency recorded from an individual PV + and SST + INs (paired *t*-test). Significant difference is marked *p < 0.05, **p < 0.01, ***p < 0.001; NS, non-significant.

Considering that brief NMDA application (3 min., 20 µM) evoked iLTP in CA1 pyramidal neurons^12^, we tested whether this protocol could induce GABAergic plasticity in PV + and SST + INs in this hippocampal region. We found that in the case of SST + INs NMDA treatment resulted in a clear mIPSC amplitude potentiation which developed in time reaching a highly significant difference with respect to baseline recordings (SST + INs: 122 ± 5%, n = 9, relative to baseline, before NMDA: 45.3 ± 3.8 pA, 16–18 min. after the end of NMDA application: 54.3 ± 4 pA, *p* = 0.003, Fig. 4a–d). Interestingly, in contrast to SST + INs for both types of PV + INs (FS and nFS) NMDA treatment resulted in a decrease in mIPSCs amplitudes. Since there was no significant difference in these effects in the two PV + INs types the data were pooled (PV + INs: 85 ± 2%, n = 12; before NMDA: 53.6 ± 6.3 pA, after NMDA: 45.3 ± 5.2 pA, *p* < 0.001; Fig. 4a–d). In addition, NMDA treatment resulted in a trend toward decreased mIPSC frequency in SST + INs (90 ± 5%, n = 9, before NMDA: 1.70 ± 0.42 Hz, after NMDA: 1.56 ± 0.41 Hz, *p* = 0.055, Fig. 4e–f) and in PV + INs a significant decrease of this parameter was observed (78 ± 7.2%, n = 12, before NMDA: 2.03 ± 0.06 Hz, after NMDA: 1.58 ± 0.16 Hz, *p* = 0.014, Fig. 4e–f).

**Figure 5:**
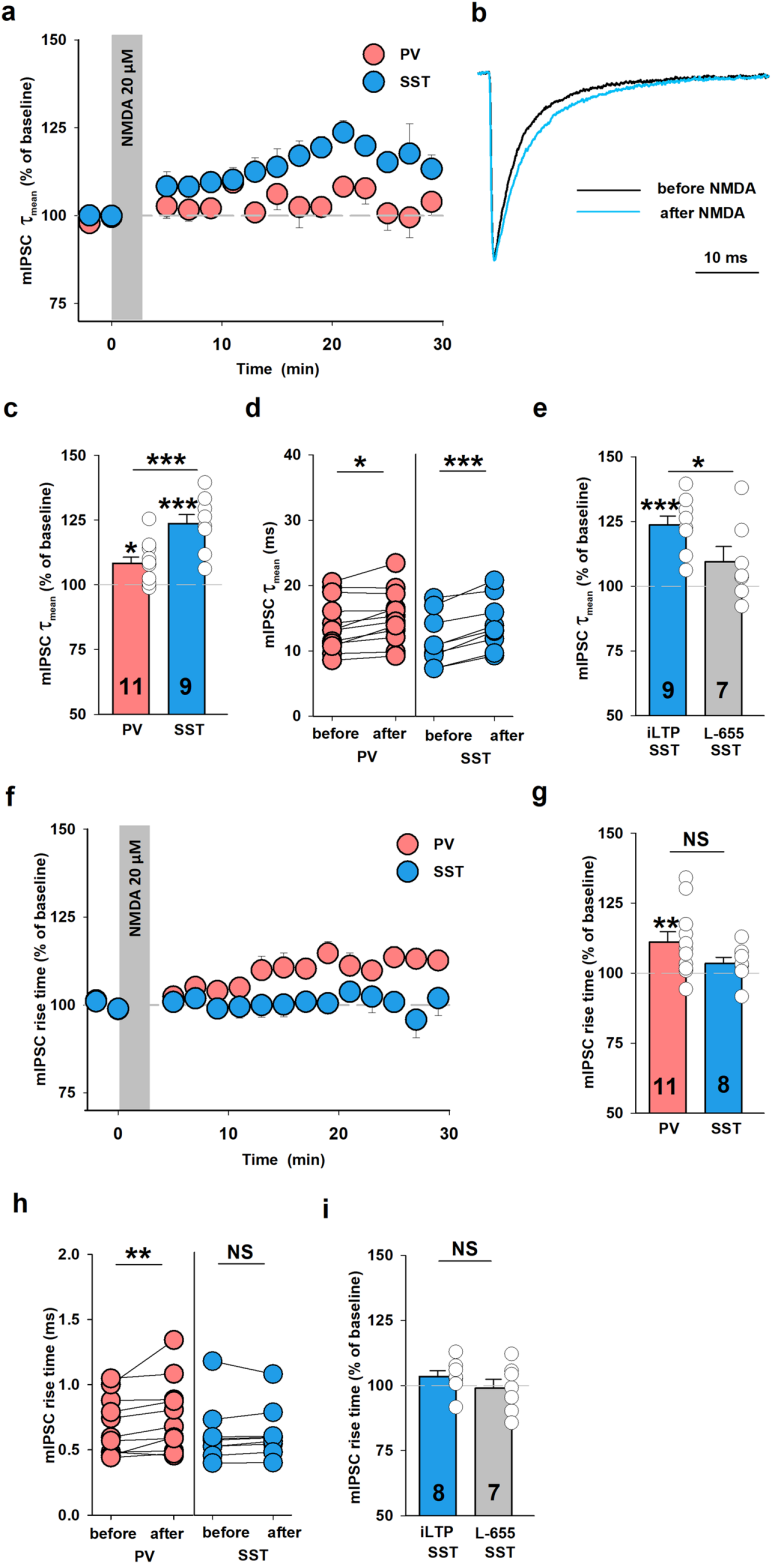
The α5-GABA_A_Rs subunit is required in NMDAR-dependent plasticity in SST + INs. (**a**) Time courses of mIPSC decay τ_mean_ after induction of iLTP with NMDA in SST + INs (blue) and iLTD in PV + INs (coral). The application of NMDA is marked in the gray bar. (**b**) Examples of averaged and normalized traces before (black) and after (blue) NMDA treatment showing the difference in the decay time course. (**c**) Statistics for changes in mIPSC decay τ_mean_ in PV + and SST + INs groups relative to baseline (asterisks above bins). Additional test for comparison between PV + and SST + INs:* t*-test, *p* = 0.001. (**d**) Absolute values of mean mIPSC τ_mean_ in individual experiments before and 16–18 min. after the end of NMDA (3 min.) administration in PV + INs and SST + INs (paired *t*-test). (**e**) Summary plot of mIPSC decay τ_mean_ changes 16–18 min. after the end of NMDA application (relative to baseline) in control group (blue bar) and in L-655,708-treated slices (50 nM, gray bar) in SST + INs. Note that prolongation of mIPSC decay time constant associated with iLTP induction was significantly reduced in the presence of α5-GABA_A_R inverse agonist (asterisk for *t*-test comparison between the two groups). (**f**) Time courses of mIPSC rise time changes after NMDA stimulation in PV + and SST + INs. (**g**) Mean value of mIPSC rise time after NMDA application in PV + and SST + INs groups relative to baseline. Asterisks indicate increase in mIPSC rise time relative to baseline in PV + INs. Additional test:* t*-test, *p* = 0.125. (**h**) Absolute values of mIPSC rise times measured before and 16–18 min. after iLTD (coral, PV + INs) or iLTP induction (blue, SST + INs). Paired *t*-test with Wilcoxon Signed Rank Test. (**i**) Summary plot of mIPSC rise time changes 16–18 min. after the end of NMDA administration in control condition (iLTP, blue) and in the presence of L-655,708 gray bar (both relative to baseline, *t*-test). *p < 0.05, **p < 0.01, ***p < 0.001; NS, non-significant.

Kinetic analysis revealed that brief NMDA application resulted in a marked prolongation of mean decay time constant (τ_mean_) of mIPSC recorded from SST + interneurons (124 ± 3%, n = 9, relative to baseline, before NMDA: 11.6 ± 1.3 ms, after NMDA: 14.1 ± 1.3 ms, *p* < 0.001, Fig. 5a–d). Also in PV + INs, NMDA treatment prolonged the mIPSC decay, but this effect was much smaller than in SST + although it was statistically significant (PV + : 108 ± 2%, n = 11; relative to baseline, before NMDA: 14.2 ± 1.2 ms, after NMDA: 15.3 ± 1.3 ms, *p* = 0.011, Fig. 5a–d). In the case of SST + INs, the mIPSC rise time was not significantly affected (rise time: 103 ± 2%, n = 8, relative to baseline, before NMDA: 0.61 ± 0.08 ms, after NMDA: 0.63 ± 0.07 ms, *p* = 0.195, Fig. 5 f–h) while for PV + INs NMDA treatment resulted in a slight but significant slow-down of mIPSC onset kinetics (rise time: 111 ± 4%, n = 11; relative to baseline, before NMDA: 0.77 ± 0.1 ms, after NMDA: 0.87 ± 0.1 ms, *p* = 0.005, Fig. 5f–h).

Prolongation of the IPSC decay kinetics has been observed in chemically induced input-specific iLTP onto pyramidal cells ^12,28^ and TBS-induced form of GABAergic plasticity ^10^ and this effect was attributed to enrichment of postsynaptic densities with α5 subunit-containing GABA_A_Rs. To test whether this mechanism is involved in the case of our model, we have applied L-655,708, a specific α5 subunit-containing GABA_A_Rs inverse agonist (50 nM ^29^) 10 min. after plasticity induction with NMDA. In SST + INs group considered in the statistics, within 10 min. after NMDA application increase in both mIPSC amplitude and τ_mean_ was observed (data not shown). However, we found that in the interneurons in which NMDA induced an increase in decay time constant, mIPSC decay markedly accelerated after L-655,708 administration (τ_mean_ 16–18 min. after the end of NMDA application—iLTP induction.: 124 ± 3%, n = 9, relative to baseline; τ_mean_ 16–18 min. after iLTP induction in the presence of L-655,708: 110 ± 6%, n = 7, relative to baseline, *p* = 0.045, Fig. 5e). The incomplete return of τ_mean_ to baseline values resulted most likely from the fact that 50 nM concentration of L-655,708 did not assure a full block of the α5 subunit-containing receptors, but a higher dose of this blocker could not be used as it could block also other GABA_A_R subtypes ^29^. In addition, the mIPSC rise time was unaffected by L-655,708 (rise time 16–18 min. after NMDA treatment: 103 ± 2%, n = 8, relative to baseline; 16–18 min. after iLTP induction in the presence of L-655,708: 98.9 ± 3%, n = 7, 16–18 min., relative to baseline, *p* = 0.275, Fig. 5i). Consistently, application of L-655,708 led to impairment of NMDA-induced iLTP manifested as increased amplitude (iLTP: 122 ± 5%, n = 9, L-655,708: 92 ± 3%, n = 9, *p* < 0.001, Supplementary Fig. 2a) but frequency remained unaffected (iLTP: 90 ± 5%, n = 9, L-655,708: 92 ± 11%, n = 9, p = 0.87, Supplementary Fig. 2b) in hippocampal SST + interneurons. In contrast to SST + INs, L655,708 did not affect NMDA-dependent iLTD in PV + INs (no effect on mIPSC amplitudes, data not shown) and therefore we have not further analyzed the impact of this compound on mIPSC kinetics. We also did not observe any effect of this α5-GABA_A_Rs inverse agonist on mIPSC parameters (amplitude, frequency, rise time and mean decay time constant) during the baseline recordings for SST + interneurons (data not shown).

In summary, we established that in SST + INs an iLTP is taking place that depends on the α5-GABA_A_Rs incorporation into inhibitory synapses whereas in PV + INs the same protocol led to mIPSC amplitude reduction.

### α5β1 integrins are involved in NMDAR-dependent iLTP in SST + INs

**Figure 6:**
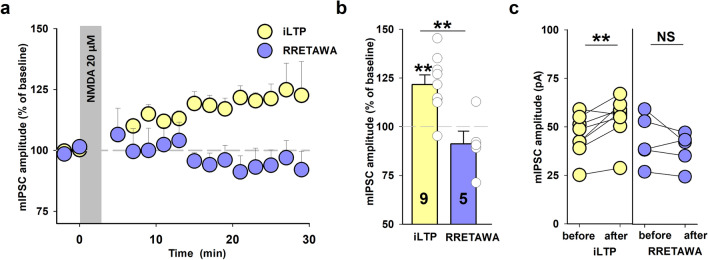
Administration of α5β1 integrin occludes NMDAR-dependent iLTP in SST + INs. (**a**) Time course of mIPSC amplitude after iLTP induction with NMDA (gray bar), recorded from control slices (yellow) and in the presence of RRETAWA peptide (0.15 mM, purple). (**b**) Statistics for iLTP magnitude 16–18 min. after the end of NMDA application in control conditions (yellow bar) and RRETAWA-treated slices (purple bar, asterisk for *t*-test comparison between the two groups). Note that the induction of NMDAR-dependent iLTP in the presence of RRETAWA was abolished. Asterisks above bin show increase in mIPSC amplitude (relative to baseline). (**c**) Absolute value of mean mIPSC amplitudes in individual recordings before and 16–18 min. after iLTP induction in control and RRETAWA-treated group (paired *t*-test). Every significant difference is marked by **p < 0.01; NS, non-significant.

As described above, we found that a synthetic cyclic peptide RRETAWA (0.15 mM) which interferes with α5β1 integrins, caused an increase in mIPSC amplitude recorded from SST + INs (Fig. 3a–d) and a similar effect was also shown for pyramidal neurons^30^. To elucidate whether α5β1 integrins might occlude NMDA-induced iLTP, we applied RRETAWA 10 min. before NMDA administration and found that the application of this peptide completely abolished this type of plasticity in SST + INs (iLTP: 122 ± 5%, n = 9, RRETAWA treatment: 91 ± 7%, n = 5, *p* = 0.003, 16–18 min. after the end of NMDA application, Fig. 6a–c). Moreover, induction of NMDAR-dependent iLTP in the presence of RRETAWA did not affect the mIPSC frequency in SST + INs (RRETAWA: 92 ± 13%, n = 5, relative to baseline, *p* = 0.707; Supplementary Fig. 1b) which is not unexpected as neither RRETAWA alone nor induction of iLTP with NMDA in the absence of this peptide affected the mIPSC frequency (Fig. 3e–f, Fig. 4e–f). We conclude that occlusion NMDAR-dependent iLTP by RRETAWA provides evidence that α5β1integrins play a key role in this type of heterosynaptic GABAergic long-term plasticity in the SST + INs.

## Discussion

The major finding of the present study is that GABAergic synaptic transmission shows various types of plasticity in the inhibitory CA1 hippocampal interneurons in a cell-specific manner. Thus, previously described inhibitory plasticity at the principal cells^10,12,16,17,30^ is most likely accompanied by finely tuned plastic changes occurring at distinct types of GABAergic interneurons controlling the activity of pyramidal cells and of the local networks.

### RGD-binding integrins are involved in the plasticity of GABAergic transmission onto interneurons

We found that administration of the GRGDSP peptide led to potentiation of inhibitory transmission in FS PV + and SST + INs, while no effect was observed in nFS cells, thus, for RGD-binding integrins, the potentiating effect was predominant. This finding appears particularly interesting in the light of our recent results that administration of GRGDSP peptide resulted in depression of GABAergic transmission recorded from pyramidal cells in CA1 region^30^. Thus, as a result of plasticity mediated by RGD-binding integrins, the pyramidal cells could be partially disinhibited as the PV + and SST + interneurons innervating the principal cell both perisomatically and at distal dendrites would be functionally weakened by enhanced inhibitory drive. The mechanism whereby RGD-binding integrins give rise to different forms of inhibitory plasticity in distinct neurons is not clear but different expression of these adhesion proteins in diverse types of cells or in distinct areas of a specific cell (e.g. at different synapses) is an obvious possibility. As already mentioned, broad spectrum integrin ligand RGD binds to α5β1, α8β1 and to the αv-containing integrins^22,31^. It has been shown that integrins containing β1 and β3 subunits colocalized with GABAergic synapses at pyramidal neurons in the CA1 area^30^. Besides pyramidal cells, expression of α1–α5, β1, β3–β5 integrin subunits were observed in astrocytes and interneurons within CA3-CA1 pathway^32^ and α5β1 type was found also in dopaminergic neurons from mesencephalic and striatal cultures^33^. Interestingly, α3- (but not α5- and αv-) subunits were found postsynaptically at GABAergic synapses of cerebellar Purkinje neurons and α3β1integrins were implicated in regulating rebound inhibitory potentiation in contrast to RGD-binding integrins^34^. Moreover, disruption of the interaction between extracellular matrix and integrins by RGD peptide enhanced GABAergic tonic currents and application of fibronectin (ligand mainly for α5β1) decreased amplitude of GABA_A_-evoked currents measured from dentate gyrus granule cells^35^. It is worth mentioning that RGD-binding integrins are also involved in regulation of plasticity at glycinergic synapses which are closely related to the GABAergic ones as both of them contain large amounts of gephyrin and GABA_A_R and glycine receptors are structurally similar belonging to the cys-loop superfamily. It has been shown that at glycinergic synapse, administration of RGD peptide increased the number of glycine receptors, leading to enhancement of synaptic strength ^13^ similar to our observations for SST + and PV + INs.

### Cell-specificity of GABAergic plasticity is associated with integrin-dependent mechanisms

Interestingly, when using cyclic synthetic peptide (RRETAWA) that blocks α5β1integrin-fibronectin interaction, a potentiation of mIPSCs in SST + INs and a reduction in FS PV + cells was observed (no effect in nFS PV +). This result might look surprising as RGD also affects the α5β1integrin and for this peptide a potentiating effect on mIPSC amplitude was observed in FS PV + INs. Notably, neither RRETAWA nor RGD had any effect in the case of nFS PV + INs and both peptides potentiated mIPSC amplitudes in SST + INs. It is also worth noting that in the case of NMDA induced plasticity (Fig. 4), mIPSCs were enhanced in SST + and reduced in PV + INs (but in FS and nFS the effect was qualitatively the same) showing a similarity to RRETAWA-induced plasticity although in the case of this peptide no effect was found for nFS PV + INs. Thus RRETAWA and RGD, to some extent, showed similar actions but in the case of FS PV + INs opposite effects were observed. This difference is not surprising as RGD affects a larger spectrum of integrins than RRETAWA and diverse expression of various integrins types in the considered INs and the specific types of synaptic contacts is most likely responsible for these divergences. Future studies focusing on the input specificity of inhibitory synaptic plasticity in relation to different integrins involvement should be explored. It is interesting to note that in our recent study Wiera et al., (2022) we found that whereas application of RGD depressed GABAergic transmission recorded from pyramidal neurons, RRETAWA potentiated it. Therefore, whereas observed here effect of RRETAWA in SST + INs was similar to that described in principal cells by Wiera et al., (2022), impact of RGD in PV + and SST + INs tended to be the opposite with respect to that in pyramidal cells^30^. Again, we may attribute these diverging effects to different sets of integrins involved and thereby to different intracellular molecular modulatory pathways.

It seems particularly interesting that NMDA-induced iLTP in SST + INs is associated with a prolonged mIPSC decay due to incorporation of α5-GABA_A_Rs into synapses. This finding is consistent with recent reports showing a slow-down of mIPSC decaying phase in pyramidal neurons after NMDA-induced iLTP^12^ and in the wake but not sleep states^28^ while in the latter study the same approach as in the present report (L-655,708 blocker of α5-GABA_A_Rs) was used. Generally, it is now well established that α5-GABA_A_Rs contribute to slow GABAergic synaptic currents^36,37^ and the picture emerges that α5 subunit is crucial in synaptic plasticity dependent on the synapse/cell-type^10,28,29^. In particular, it is noteworthy that in the study by Davenport et al., (2021) it was shown that induction of excitatory LTP drives α5-GABA_A_Rs into inhibitory synapses exemplifying thus a heterosynaptic cross-talk between excitatory and inhibitory synapses^10^.

The opposite signs of GABAergic plasticity observed here in PV + and SST + INs in the case of RRETAWA administration or transient treatment with NMDA appear to be particularly interesting. As already mentioned, these interneurons innervate separate and distant regions of pyramidal neurons being involved in distinct computational stages performed by the local neuronal network. Indeed, PV + basket cells and O-LM and bistratified cells (both SST +) are being activated at distant phases of the theta cycle—PV + basket cells close to the peak while SST + INs—at the trough. Royer et al., (2012) have found that optogenetic silencing of PV + or SST + cell clearly affected the pyramidal neuron’s firing rate and, within a given place field, the effect of PV + INs was highest when the animal entered the place field, and decreased when the animal proceeded forwards while the opposite trend was observed for SST + INs^38^. We can thus speculate that opposite trends in GABAergic plasticity in PV + and SST + INs could be involved in optimizing the network functioning associated with animal behavior.

A prominent type of GABAergic plasticity considered here is heterosynaptic, resulting from a transient activation of NMDAR^12,16,17^, and therefore its molecular mechanism might depend on the involvement of integrins in regulating the glutamatergic drive. Pharmacological inhibition of α5- and β1 subunit-containing integrins by using antibodies and synthetic peptide was shown to abolish LTP of excitatory transmission^39^. It was demonstrated that RGD-binding integrins or fibronectin were involved in induction of robust GluN2A and GluN2B tyrosine phosphorylation of NMDA receptor, giving rise to an increase in NMDA receptor-mediated synaptic responses^40^. Michaluk et al., (2009) have shown that enzymatic activity mediated by MMP-9 increased NR1-NMDAR surface trafficking and proposed a mechanism dependent on β1 subunit-containing integrins ^41^. Juhasz et al., (2008) used in vivo recordings and found a dose-dependent effect of RGD peptide on responses mediated by NMDA or AMPA receptors^42^. In an electrophysiological study, Chan et al. (2006) have shown that in a model in which β1 integrin was knocked out in forebrain excitatory neurons, an impaired synaptic transmission through AMPA receptors and weakened NMDAR-dependent long-term potentiation were observed^15^. In the context of integrin-dependent modulation of glutamatergic (especially NMDAR-dependent) drive it is worth mentioning that in the present study application of RRETAWA peptide interfering with α5β1 integrins prevented NMDAR-dependent induction of iLTP in SST + INs similar to iLTP in pyramidal cells described in our recent report^30^. It may be hypothesized that this effect of integrins on iLTP might be, at least in part, due to their action on NMDARs. On the other hand, in our recent study we have reported that fibrinogen-induced iLTD onto pyramidal cells required the activity of calcineurin but not of Src, CaMKII or PKC kinase^30^. We may thus speculate that NMDA-induced iLTD at inhibitory synapse onto PV + INs described here might involve analogous molecular player. Moreover, it is also noteworthy that integrins may play a role in structural plasticity. Shi and Ethell (2006) have reported that RGD treatment resulted in alteration of spine shape^43^. This effect was associated with integrin-dependent actin reorganization which could be partially prevented by function-blocking antibodies against β1and β3 integrins. Involvement of integrins in the spine shape modulation could be relevant to GABAergic synaptic plasticity as in a population of spines, glutamatergic synapses are accompanied by GABAergic ones^4^. It has been also shown that NMDA-induced cytoskeletal disassembly is dependent on integrins^44^.

In conclusion, our results reveal that plasticity of inhibitory synapses at considered GABAergic cells shows interneuron-specificity and strongly rely on the activity of distinct types of integrins. This study provides the first evidence that disinhibition, a key network mechanism, may be a highly plastic process depending on interneuron type and integrins’ activity.

## Materials and methods

### Animals

All animals were kept in Experimental Animal House of the Wroclaw Medical University on a natural light/dark cycle. We carried out our experiments on genetically modified male and female mice that express fluorescence reporter in a specific type of interneurons-Parvalbumin (PV +) INs and Somatostatin (SST +) INs. Homozygous knock-in mice that express Cre recombinase: PV-Cre (JAX 017320) and SST-Cre (JAX 028864) with Rosa26-tdTomato reporter mice (Ai14, JAX 007914) were used which has been approved by Polish Ministry of Environment. The study was reported in accordance with ARRIVE guidelines.

### Slice preparation

Under isoflurane anesthesia, aged postnatal day 18–23 mice were decapitated using procedures in accordance with the Polish Animal Protection Act (Act of 15 January 2015, changed 17 November 2021; directive 2010/63/EU). Removed brains were submerged in ice-cold oxygenated ACSF containing (in mM): 119 NaCl, 26.3 NaHCO_3_, 11 glucose, 2.5 KCl, 1 NaH_2_PO_4_, 1.3 MgSO_4_, 2.5 CaCl_2_ (pH 7.4). Subsequently, 350 μm thick transverse hippocampal slices were prepared using a vibratome (VT1200S, Leica) and recovered for at least 1.5 h (room temperature 21–23 °C, the same solution described above) before being used for electrophysiological recordings.

### Electrophysiological recordings and data analysis

After incubation, single hippocampal slice was transferred to a submersion-type recording chamber and superfused with ACSF at RT at 2.5–3.5 ml/min, bubbled with 95% O_2_-5% CO_2_. Whole-cell patch-clamp recordings were made from interneurons located in CA1 region of the hippocampus using borosilicate patch pipettes with a resistance of 3–4 MΩ filled with the intracellular solution containing (in mM): 10 potassium gluconate, 125 KCl, 1 EGTA, 10 HEPES, 4 MgATP, and 5 sucrose, pH 7.5, 285 mOsm. PV + INs and SST + INs were identified based on tdTomato expression, visualized by fluorescence microscopy. Recordings of mIPSC were made at a holding potential − 70 mV using the MultiClamp 700B amplifier and Digidata 1550B digitizer (Molecular Devices). After stable baseline measurement, with glutamate receptors blocked by DNQX (20 µM) and Na^+^ channels inhibited by 1 µM TTX (at least 12 min.), plasticity was evoked by transient exposure to NMDA (3 min., 20 µM) and then monitored for at least 30 min. after NMDA wash out. Input resistance was measured throughout the experiments and data were discarded from analysis if changed by more than 20%. mIPSC were analyzed for amplitude, frequency and kinetics using Clampfit 10.7 software (Molecular Devices) and SigmaPlot 11 software as described ^12^. The mIPSC onset kinetics was assessed as 10% to 90% rise time while decay kinetics was fitted with a biexponential function (A_1_exp(-t/τ_fast_) + A_2_exp(-t/τ_slow_), where A_1_ and A_2_ are amplitudes, τ_fast_, τ_slow_ are the time constants) and the mean decay time constant was calculated as τ_mean_ = a_1_τ_fast_ + a_2_τ_slow_, where a_1_ = A_1_/(A_1_ + A_2_) and a_2_ = A_2_/(A_1_ + A_2_). Synaptic events were detected manually and averaged over two-minute time windows and the mean values of peak amplitude, frequency, rise time and mean decay time constant were compared before and after plasticity induction. The extent of plasticity for PV + INs and SST + INs groups was calculated at the time interval 16 –18 min. after the end of the NMDA administration (lasting 3 min).

Among PV + INs two classes were considered: FS and nFS which were distinguished based on differences in the firing rate, action potential (AP) half-width and in the membrane time constant. The firing rates was measured as the number of action potentials during fourth depolarizing current step (300 ms long, 25 pA increments) counted from the threshold stimulus which was the first to evoke AP. AP half-width was estimated for the first action potential elicited by a depolarizing current pulse with the AP threshold defined as the voltage at which the slope trajectory reaches 20 mV/ms. The membrane time constant was calculated from a single exponential fit to the rising phase of response to a hyperpolarizing current step (− 25 pA). Interneurons were classified as FS INs when displayed high firing rates (range: 20–77 Hz), narrow spike widths (range: 0.43–0.87 ms) and short time constants (range: 4.3–9.68 ms). Interneurons were assigned to the nFS group if they had lower mean firing frequencies (range: 16–33 Hz), broader action potential half-widths (range: 1.15–1.78 ms) and longer time constants (range: 9.44–20.18 ms). As explained above FS INs were selected based on the firing pattern properties. It needs to be mentioned that in the CA1 area of mice hippocampus two classes of fast-spiking PV + INs are distinguished: basket and chandelier cells^4,45^. However, it was estimated that in stratum pyramidale basket cells represent 60% of PV + interneurons while chandelier cells only 15%^45^ so, it is expected that majority of cells classified as FS INs are basket cells. Importantly, some interneurons express both molecular markers (PV and SST) e.g. oriens-lacunosum moleculare INs (O-LM) with somata located in the St. oriens and bistratified INs. However, somata of bistratified INs are localized in St. oriens (24%), St. pyramidale (70%), and St. radiatum (6%)^46^. Thus, our nFS PV-positive INs group (recorded from St. pyramidale and St. radiatum) could include bistratified INs. Nonetheless, in our SST + INs group we include predominantly O-LM INs with horizontally located soma in St. oriens close to alveus.

The effect of integrins on GABAergic synaptic transmission was studied by applying the integrin-interfering peptides after baseline recordings at concentrations previously used by our group^30^. In the figures their effect is presented for the entire recording time after peptide administration (0–30 min) but in results section a numerical value obtained for the time window 16–18 min. is reported.

For all data, normality test (Shapiro–Wilk test) was performed followed by parametric or non-parametric tests (see text and figure legends for specifics). The statistical significance was determined as *p* < 0.05.

### Reagents

Reagents not listed in Table 1 were purchased from MilliporeSigma.

**Table 1:** Sources and catalog numbers of crucial reagents used.

Reagent	Source	Identifier
Tetrodotoxin	Latoxan	Cat# L8503
DNQX disodium salt	Tocris Bioscience	Cat# 2312
DMSO	Millipore sigma	Cat# D2650
Peptide GA(C)RRETAWA(C)GA	Proteogenix	Synthetized on demand
Peptide GRGDSP	Proteogenix	Synthetized on demand
Peptide GRADSP	Proteogenix	Synthetized on demand
L-655,708	Tocris Bioscience	Cat# 1327

### Ethical approval

All experiments were carried out in accordance with the Polish Animal Protection Act (Act of 15 January 2015, changed 17 November 2021; directive 2010/63/EU).

## Supplementary Information

Supplementary Information 1.

Supplementary Information 2.

Supplementary Information 3.

## Data Availability

The data and material that support the findings of this study are available upon request to the corresponding authors.
