# Supplementary figures and images for: GABAergic synapses onto SST and PV interneurons in the CA1 hippocampal region show cell-specific and integrin-dependent plasticity
Source: Sci Rep. 2023 Mar 28;13:5079. doi: 10.1038/s41598-023-31882-4 (PMC10050003; doi:10.1038/s41598-023-31882-4)

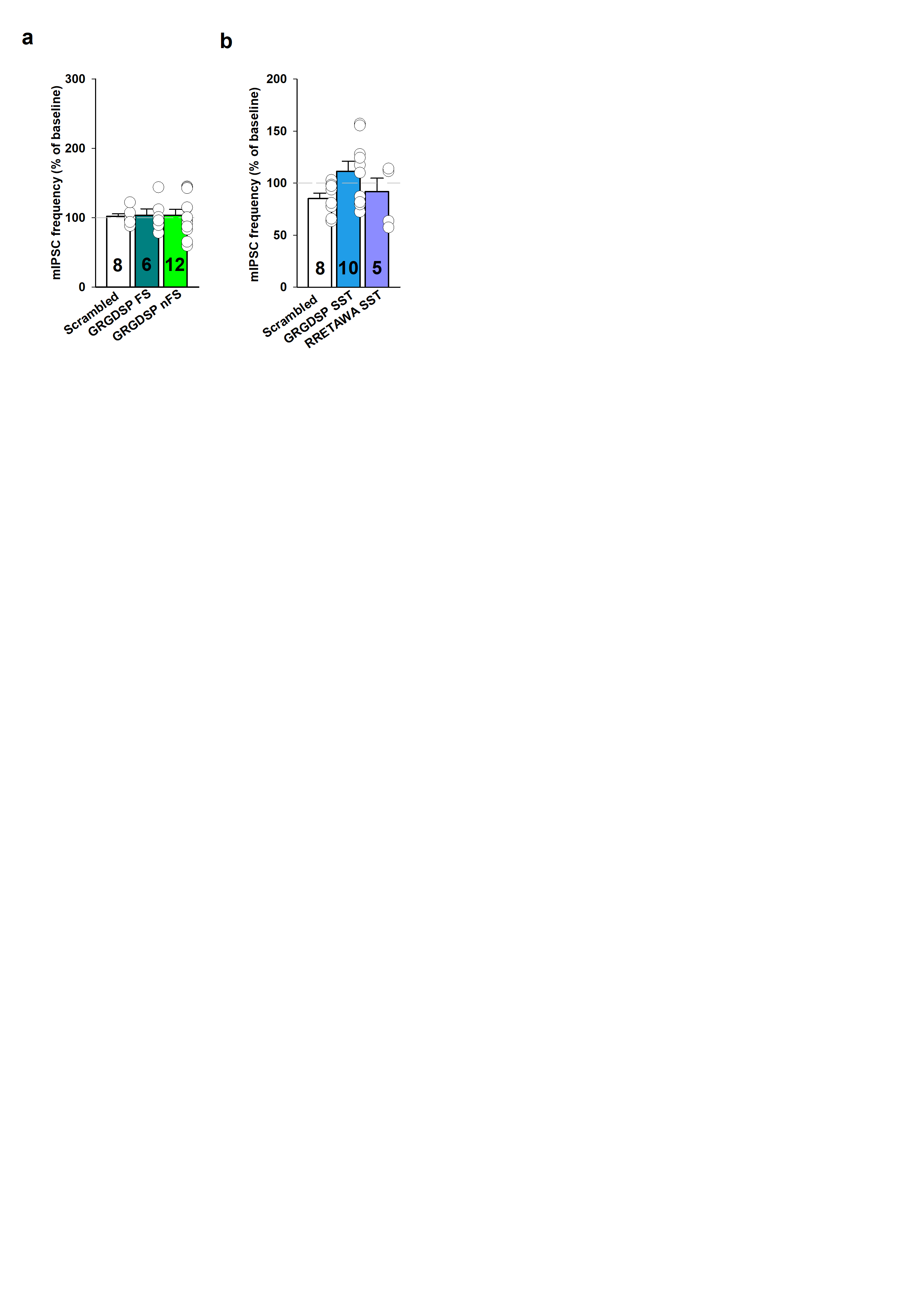

Supplement: Supplementary file 1 — Supplementary Information 1. [file 41598_2023_31882_MOESM1_ESM.tif]

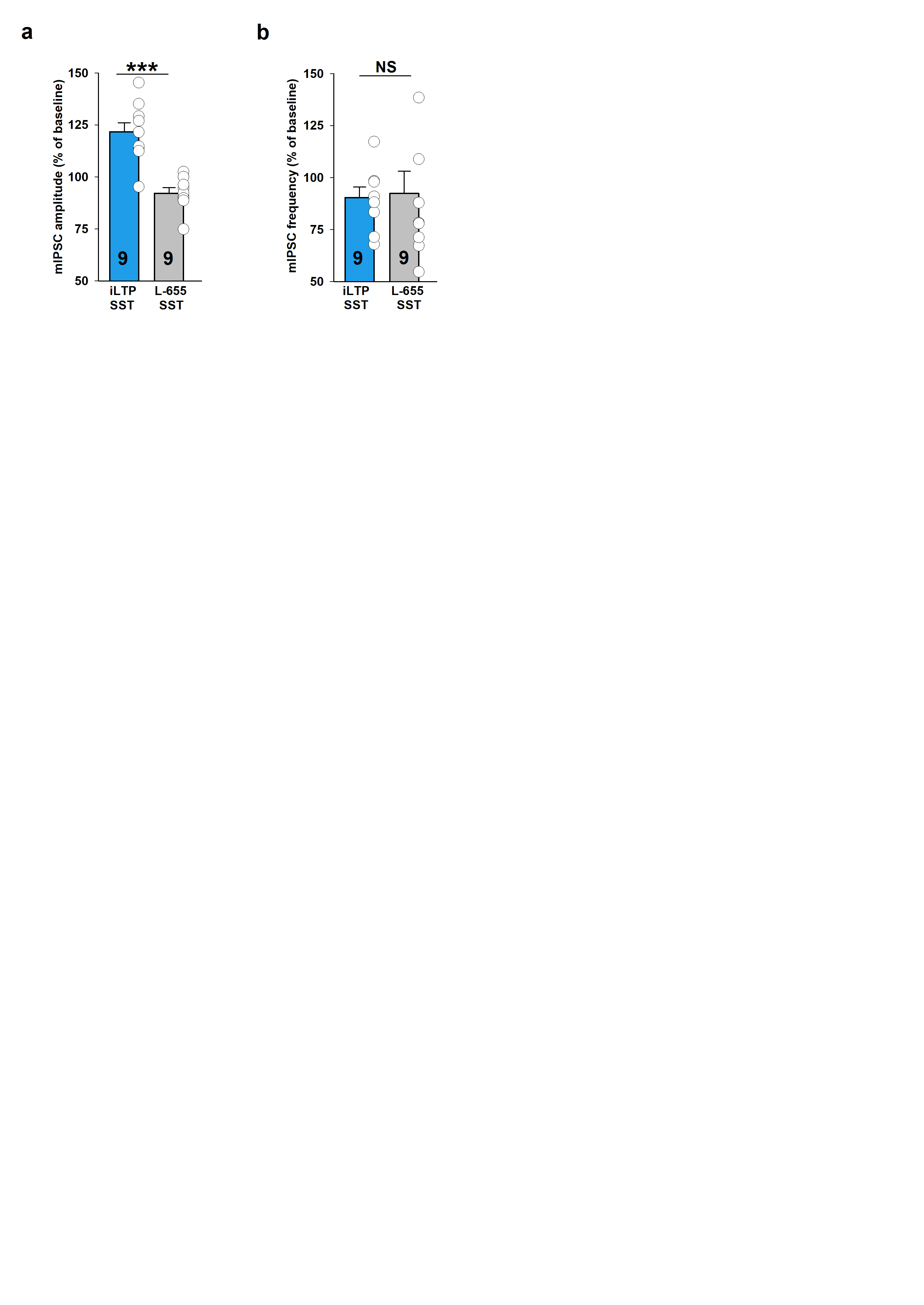

Supplement: Supplementary file 2 — Supplementary Information 2. [file 41598_2023_31882_MOESM2_ESM.tif]
