# Supplementary material for: GABAergic synapses onto SST and PV interneurons in the CA1 hippocampal region show cell-specific and integrin-dependent plasticity
Source: Sci Rep. 2023 Mar 28;13:5079. doi: 10.1038/s41598-023-31882-4 (PMC10050003; doi:10.1038/s41598-023-31882-4)
Supplement: Supplementary file 3 — Supplementary Information 3. [file 41598_2023_31882_MOESM3_ESM.docx]

**Supplementary**  **Fig. 1 The impact of integrin-binding peptides on mIPSC frequency**

**a-b** Summary of mIPSC frequency change 16-18 min. after application of integrin-binding peptides relative to baseline. **a** Scrambled peptide effect (GRADSP, white), GRGDSP in FS (dark green) and nFS (light green) PV+ INs groups **b** Scrambled peptide effect (GRADSP, white), GRGDSP (blue) and RRETAWA (purple) in SST+ INs.

**Supplementary**  **Fig. 2 Blocker of α5 subunit-containing GABA_A_Rs, L-655,708 abolishes increase in mIPSC amplitude after NMDA treatment in SST+ INs**

**a-b** The effect of L-655,708 (50 nM) application 10 min. after NMDA administration on mIPSC amplitude (**a**) and frequency (**b**) measured 16-18 min. post iLTP induction (gray bars compared to statistics in control conditions – blue bars). Note that increase in amplitude associated with iLTP is abolished in the presence of α5-GABA_A_Rs inverse agonist. (**a-b**) *t*-tests for comparison between controls and L-655-708 treated groups. ***p < 0.001; NS, non-significant.
